# The Role of Neurogenic Inflammation in Blood-Brain Barrier Disruption and Development of Cerebral Oedema Following Acute Central Nervous System (CNS) Injury

**DOI:** 10.3390/ijms18081788

**Published:** 2017-08-17

**Authors:** Annabel J. Sorby-Adams, Amanda M. Marcoionni, Eden R. Dempsey, Joshua A. Woenig, Renée J. Turner

**Affiliations:** Adelaide Medical School and Adelaide Centre for Neuroscience Research, Faculty of Health and Medical Sciences, The University of Adelaide, Adelaide SA 5005, Australia; annabel.sorby-adams@adelaide.edu.au (A.J.S.-A.); amanda.marcoionni@student.adelaide.edu.au (A.M.M.); edendempsey19@gmail.com (E.R.D.); josh.woenig@adelaide.edu.au (J.A.W.)

**Keywords:** substance P, calcitonin gene-related peptide, neuropeptides, neurogenic inflammation, cerebral oedema, stroke, traumatic brain injury, tachykinin, blood-brain barrier

## Abstract

Acute central nervous system (CNS) injury, encompassing traumatic brain injury (TBI) and stroke, accounts for a significant burden of morbidity and mortality worldwide, largely attributable to the development of cerebral oedema and elevated intracranial pressure (ICP). Despite this, clinical treatments are limited and new therapies are urgently required to improve patient outcomes and survival. Originally characterised in peripheral tissues, such as the skin and lungs as a neurally-elicited inflammatory process that contributes to increased microvascular permeability and tissue swelling, neurogenic inflammation has now been described in acute injury to the brain where it may play a key role in the secondary injury cascades that evolve following both TBI and stroke. In particular, release of the neuropeptides substance P (SP) and calcitonin gene-related peptide (CGRP) appear to be critically involved. In particular, increased SP expression is observed in perivascular tissue following acute CNS injury, with the magnitude of SP release being related to both the frequency and degree of the insult. SP release is associated with profound blood-brain barrier disruption and the subsequent development of vasogenic oedema, as well as neuronal injury and poor functional outcomes. Inhibition of SP through use of a neurokinin 1 (NK1) antagonist is highly beneficial following both TBI and ischaemic stroke in pre-clinical models. The role of CGRP is more unclear, especially with respect to TBI, with both elevations and reductions in CGRP levels reported following trauma. However, a beneficial role has been delineated in stroke, given its potent vasodilatory effects. Thus, modulating neuropeptides represents a novel therapeutic target in the treatment of cerebral oedema following acute CNS injury.

## 1. Introduction

Acute central nervous system (CNS) injury, encompassing traumatic brain injury (TBI) and stroke, is a leading cause of death and disability worldwide [1,2,3]. TBI is the leading cause of death in those under 45 years of age, accounting for upwards of 10 million deaths and hospitalisations worldwide each year [1,2]. Stroke affects in excess of 15 million people globally each year, of which six million die, and five million are left permanently disabled [3]. For those who survive the initial injury, many face long-term disability and dementia, requiring extensive rehabilitation and assistance with daily living activities [2,4]. Indeed, there is an estimated 15 million people living with the sequelae of TBI and over 30 million stroke survivors with significant disabilities [1,4]. Thus, the socioeconomic burden of acute CNS injury is immense, costing the global healthcare system in excess of US $200 billion dollars each year, which does not even begin to account for the extensive cost to individuals quality of life, caregivers and communities [5]. Thus, new treatments are urgently required to reduce patient morbidity and mortality following acute CNS injury. Neurogenic inflammation has been proposed as a novel target in treating acute CNS injury. This neutrally-elicited process is characterised by the release of neuropeptides which induces vasodilation, increased microvascular permeability and tissue swelling. Thus, this review will provide an overview of studies supporting a role for neurogenic inflammation in increased blood-brain barrier (BBB) permeability, cerebral oedema formation and development of functional deficits following acute CNS injury.

### 1.1. Traumatic Brain Injury

TBI results from acceleration/deceleration forces that produce rapid movement of the brain within the skull, or from the head impacting with an object. The most common causes of TBI are motor vehicle accidents, motorcycle and pedestrian injuries [6], with falls the most common cause in the elderly population [7]. The resultant pattern of injury depends upon a number of factors including: the nature of the initiating force, site of impact and the direction/magnitude of the impact. Injury to brain tissue in TBI can be divided into two main categories: primary injury and secondary injury. Primary injury occurs at the moment of injury and encompasses the shearing/tearing/stretching of axons, known as axonal injury, in addition to lacerations, contusions and haemorrhages. Following this is secondary injury, which is initiated by the primary insult and evolves over the hours, days to weeks following the traumatic event and is well documented to exacerbate brain injury and worsen outcome following TBI [8].

### 1.2. Stroke

Stroke results from an interruption in cerebral blood flow (CBF), most commonly due to the permanent or transient occlusion of a cerebral artery [9]. This sudden and profound reduction in CBF restricts the supply of vital oxygen and nutrients to the brain tissue, leading to ischaemia, cell injury and ultimately cerebral infarction. Ischaemic stroke is the most common type, accounting for approximately 85% of all strokes and typically occurs in the setting of atherothrombosis [10]. The remaining 15% are classified as haemorrhagic and arise due to the rupture of a cerebral artery [10]. The infarct that results from the cerebral ischaemia is comprised of two distinct regions: the core and the penumbra. The infarct core represents an area of tissue that has succumbed to the ischaemia and undergone rapid neuronal cell death [11]. Surrounding the core is the penumbral tissue, neurons in this area are compromised by the reduction in CBF but are still viable. If ischaemia continues then neurons within the penumbra may progress to become irreversibly damaged and undergo cell death, thereby increasing infarct size. However, with adequate and timely reperfusion neuronal loss may be reduced and functional outcome improved and therefore the penumbra is of the greatest clinical interest, with the goal of thrombolysis therapy with tissue plasminogen activator (tPA) to salvage penumbral tissue [12]. As in TBI, the injury that occurs following stroke can be divided into primary and secondary injury components. Within minutes of cerebral ischaemia onset cell death occurs, this is the primary injury, following which the secondary injury cascades are initiated, which lead to infarct expansion and worsened outcomes following stroke [9].

## 2. Secondary Injury

Both TBI and stroke have a primary and secondary injury component and although the primary injury mechanisms are somewhat different, there are many shared features of the secondary injury cascades. Such secondary injury involves a cascade of injury pathways that exacerbate tissue damage and worsen outcome including: inflammation, excitotoxicity, oxidative stress, loss of ion homeostasis and increased BBB permeability, amongst many others [8,13,14]. Indeed, much of the death and disability associated with acute CNS injury is attributable to the development of secondary injury processes that are initiated by the primary injury and evolve over time in the days to weeks following the initial insult [15], in particular alterations in BBB permeability and the subsequent development of life-threatening cerebral oedema and its associated complications.

## 3. The Blood-Brain Barrier

Injury to the BBB and alterations to BBB permeability are key secondary injury processes following both TBI and stroke. The BBB is a highly specialised, semi-permeable barrier existing between the brain and blood that serves to maintain homeostasis of the cerebral microenvironment by restricting the passage of compounds and toxins into the CNS [16]. Structurally, the barrier comprises an array of components including endothelial cells with tight junctions (TJ), adherens junctions, astrocytes, pericytes, and the basement membrane [16]. Together, these components provide the structural integrity required to enable the barrier to maintain fundamental roles including: supplying the brain with essential nutrients such as oxygen and glucose, mediating the efflux of waste products, and facilitating the movement of nutrients and plasma proteins [17].

Under normal physiological conditions, nutrients and plasma proteins cross the BBB via two main transport mechanisms: paracellular transport and transcellular transport. Paracellular transport involves the passage of small solutes (<800 Da) between endothelial cells, as facilitated by TJs [18]. Conversely, transcellular transport is used by large plasma proteins, such as albumin, to cross the BBB by caveolae-mediated endocytosis. Vesicular trafficking of albumin occurs following budding of caveolae into vesicles that migrate to the plasma membrane to fuse and release their contents [18]. Each of these transport mechanisms is central to the maintenance and function of the barrier. Caveolae are flask-shaped plasma membrane invaginations are central in the vesicular trafficking of plasma proteins and are abundant in endothelial cells of the BBB with cholesterol and caveolin-1 as significant structural components [19]. Caveolin-1 is the integral protein essential for caveolae formation, as mice with genetic ablation of caveolin-1 lack caveolae [20], and cannot endocytose albumin from the vasculature [21].

### 3.1. Cellular Components of the BBB

Astrocytes are an integral cellular component that influence both the structure and function of the BBB. Astrocytic end-feet envelope the endothelial cells, providing structural support and enhancing the TJs in between [22]. Astrocytes are able to rapidly respond to pathological stimuli in their surrounding environment and do so through conversion to a hypertrophic state and increased expression of intermediate filaments, such as glial fibrillary acidic protein. Pericytes are the other key cellular component, which have diverse functions including: regulation of capillary haemodynamics [23], permeability of the BBB, clearance of toxic metabolites, angiogenesis and neuroinflammation [24].

### 3.2. Tight Junctions

Tight and adheren junctions form the junctional complexes that make up the BBB and are comprised of a complex network of transmembrane and cytosolic proteins, that allow TJs to seal and mediate the gate function of the BBB [25]. TJs are domains of occluded intercellular clefts, comprised of the integral membrane proteins occludin [26], claudins [27], zona occludin-1 (ZO-1) [28], and junctional adhesion molecules (JAMs) [29]. Occludin was the first TJ protein identified and is one of the main TJ components [30]. It is comprised of 9 domains, with the second extracellular domain having a pivotal role in occludin assembly and localisation into TJs [31]. This extracellular domain has also been implicated in altering TJ permeability when occludin levels decline [32]. It was previously suggested that occludin plays an important role in forming TJ-like structures [33], however TJ strands still develop in the absence of occludin [34]. Moreover, the morphology of TJs and trans-epithelial resistance in occludin-deficient mice do not differ from wild-type mice [34], suggesting that occludin is dispensible for TJ formation.

Claudins play a major role in both establishing and maintaining properties of the barrier [27,35]. Upon the discovery of claudin-1 and claudin-2, it was revealed that they, not occludin as previously thought, were the major contributor to TJ strand formation [36]. Indeed, claudin-5-deficient mice demonstrate a size-selective loosening of the BBB, whereby the movement of small molecules across the barrier via paracellular transport is upregulated [35]. When Madin-Darby Canine Kidney Epithelial (MDCK) cells, a commonly used cell line that models the epithelium, were transfected with claudin-1, there was an increase in trans-epithelial resistance and a reduction in paracellular transport. This is in contrast to what has been demonstrated in occludin-deficient mice, where no changes in transepithelial resistance or paracellular transport were observed [34].

ZO-1 belongs to the family of membrane-associated guanylate kinase (MAGUK) proteins [37] which binds to the actin cytoskeleton, stabilising the TJ and contributing to their function. ZO-1 is also integral mediating paracellular permeability [38] with dissociation of ZO-1 leading to increased BBB permeability [39]. In vitro studies have established roles for ZO-1 in endothelial cell-cell tension and recruitment of TJ proteins [38,40,41]. These various components that comprise the BBB are tightly regulated to ensure that integrity and functionality is maintained. Nevertheless, following acute CNS injury, disruption to the structure and function of these barrier components results in profound changes in BBB permeability.

## 4. BBB Disruption Following Acute CNS Injury

Despite differences in the nature of the primary injury, loss of BBB structural integrity and heightened permeability are central features of both stroke and TBI pathogenesis. Nevertheless, in both cases the degree and timing of BBB alterations are highly dependent upon the injury conditions/injury model used. The exact mechanisms by which acute CNS injury disrupts the BBB in the setting of TBI and stroke is debatable, however acute hypertension, hyperosmolar solutions classical inflammation, enhanced para/transcellular transport and enhanced activity of matrix metalloproteinases (MMPs) have all been implicated, amongst many others [42]. Such alterations in barrier permeability following acute CNS injury arise due to loss or alterations in the function of key structural and functional components [43], which has major implications for injury progression and outcome.

### 4.1. BBB Disruption Following TBI

Alterations in BBB permeability have been well-documented following TBI [44,45], with the exact temporal profile of such changes highly variable depending upon the type of injury. For example, diffuse TBI produces early permeability changes to the BBB [46], whereas focal injury may be associated with a bi-phasic profile of BBB permeability changes [47]. Rodent TBI studies have demonstrated a rapid increase of TGF-β1 expression in the injured cortex between 6 and 12 h following TBI [48]. Furthermore, in vitro studies using the hCMEC/D3 cell line supported a relationship between increasing TGF-β1 levels and enhanced paracellular permeability following TBI, mediated through a reduction in claudin-5 expression [49]. Delayed opening of the BBB following trauma has been attributed to alterations in MMP expression and activity. In particular, MMP-9 expression was significantly upregulated beginning at 4 h and persisting to 5 days post-TBI, while MMP-2 was elevated at one, three and five days following rodent TBI [50]. Indeed, similar findings have been observed in clinical TBI with levels of both MMP-2 and MMP-9 elevated in the serum of TBI patients at 24 h compared to healthy controls [51]. Such elevations in MMP levels following TBI are significant in that they precede breakdown of key TJ components and the basal lamina, in addition to stimulating the recruitment and migration of inflammatory cells [52].

### 4.2. BBB Disruption Following Stroke

Disruption to the BBB following stroke is a well-documented feature of ischaemic injury however, the timing and extent of such permeability changes vary according to the severity and duration of the ischaemic insult. Nevertheless, it is accepted that stroke is associated with both early and late alterations in BBB permeability [52], with the first alteration in BBB permeability occuring within hours of stroke onset, and the second occurring some 24–48 h later [53]. For example, experimental stroke models have revealed that heightened barrier permeability observed at 4–12 h post-stroke is attributable to elevations in caveolin-1 expression, driving changes in the transcellular pathway, followed by later disruption at 24–48 h due to dysregulation of TJ proteins occludin, claudin-5, and ZO-1, which initiates changes in the paracellular pathway [54,55]. Furthermore, increased activity of the MMP system has also been implicated in post-stroke BBB permeability changes [52]. Specifically, very early BBB permeability alterations have been attributed to the activity of MMP-2 which loosens the tight junctions [56], whereas delayed permeability changes at 4 h–4 days post-stroke are linked to profound MMP-9-mediated degradation of the basal lamina [57] and tight junction components [58,59]. Such findings are in keeping with that in clinical stroke with similar elevations in MMP levels observed in patients with ischaemic stroke [60,61,62,63].

## 5. Consequences of BBB Breakdown: Cerebral Oedema and Elevated Intracranial Pressure

The profound alterations in BBB integrity following acute CNS injury are permissive to the development of life-threatening complications such as cerebral oedema and elevated intracranial pressure (ICP) [64]. Cerebral oedema is the abnormal and excessive accumulation of fluid within the brain parenchyma [13] and the progression of cerebral oedema following cerebral infarction or trauma exerts a mechanical force of oedematous tissue onto adjacent structures, coming at great neurological expense to surrounding neurons [65,66].

### 5.1. Cerebral Oedema

**Figure 1:**
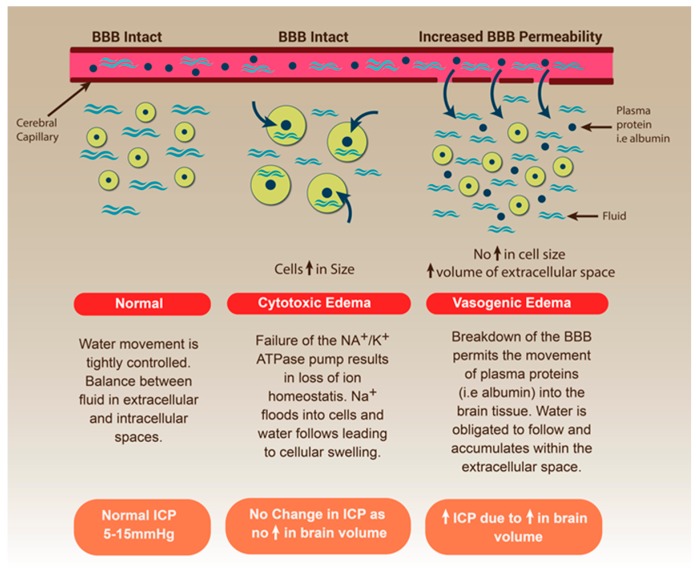
Development of cerebral oedema in acute central nervous system (CNS) injury. Note the relative size of the cells within the brain tissue and surrounding volume of the extracellular space under normal conditions and how this differs in both cytotoxic oedema and vasogenic oedema. Arrows indicate compartmental movement of water.

Cerebral oedema can be broadly divided into two categories based on the integrity of the BBB and site of fluid accumulation: cytotoxic oedema and vasogenic oedema (Figure 1) [13]. Cytotoxic oedema occurs immediately following an insult to the brain tissue and is characterised by the intracellular accumulation of fluid, particularly in the grey matter of the brain [13,67]. For example, following ischaemic stroke, deprivation of oxygen and glucose to neuronal cells as a result of vascular obstruction rapidly leads to a lack of ATP and results in irreversible cell death within minutes [67]. Similarly, the bioenergetic crisis that ensues following TBI as a result of secondary injury processes leads to a lack of ATP production. In both cases this leads to failure of the Na^+^/K^+^-ATPase pump, essential for the maintenance of ion homeostasis [68]. Such failure results in an inability to maintain ionic gradients across the membrane and leads to intracellular accumulation of sodium, creating an osmotic drive for water to move from the extracellular compartment. Such intracellular fluid accumulation leads to cellular swelling and ultimately cell rupture, which causes inflammation and collateral damage to adjacent cells [69,70].

Vasogenic oedema occurs in the setting of BBB disruption, and unlike cytotoxic oedema which occurs early following a CNS insult, typically peaks at approximately 3–5 days following the initial insult [64,67]. Endothelial dysfunction, loss of TJ integrity and enhanced transcellular transport have all been implicated in driving the abnormal extravasation of large molecules and plasma proteins, whose movement across the barrier is normally tightly regulated under physiological conditions [71]. The movement of these molecules from the intravascular compartment to the extracellular space alters the osmotic pressure, providing a driving force for the movement of water into the cerebral parenchyma [67]. The excessive and persistent movement of fluid from the blood into the brain results in the gross accumulation of water within the brain tissue, particularly within the white mater structures, thereby increasing overall brain volume and therefore increasing ICP [13]. Given that cytotoxic oedema involves only a compartmental shift of water from the extracellular to the intracellular space, and thus does not contribute to an increase in overall brain volume [71], it is vasogenic oedema that is the primary target when treating cerebral oedema.

### 5.2. Elevated Intracranial Pressure

**Figure 2:**
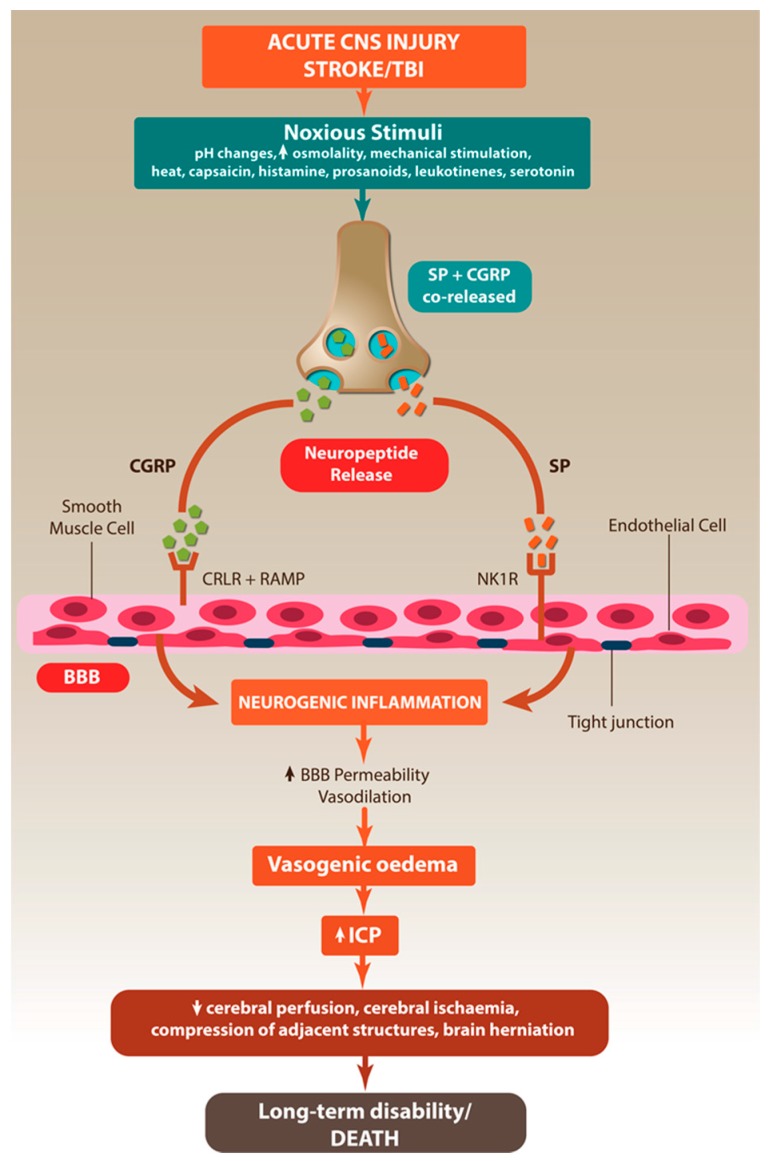
Neurogenic inflammation in acute central nervous system (CNS) injury. Acute CNS injury stimulates the release of neuropeptides, which lead to the development of neurogenic inflammation in the CNS, characterised by vasodilation, increased blood-brain barrier (BBB) permeability and cerebral oedema. Arrows indicate sequence of events following acute CNS injury.

An increase in total brain volume within the closed cavity of the skull as a result of vasogenic oedema leads to a rise in ICP (Figure 2) [72]. The development of cerebral oedema and concomitant rise in ICP is the leading cause of death in the first week following TBI and stroke, with the mortality rate of malignant cerebral oedema approaching 80% and is also a predictor of poor outcome in survivors [64,67,73].

Normal human ICP lies between 5 and 15 mmHg, and elevations in pressure upwards of 20 mmHg are associated with poor brain perfusion and an increased risk of death and disability following acute CNS injury [74,75]. The skull and underlying inelastic dura mater restrict the expansion of the brain tissue, and as such, when the volume of one of the compartments is increased (brain tissue, arterial blood, venous blood or cerebrospinal fluid) then this must be compensated for by a decrease in one of the other compartments, otherwise pressure will rise [65]. Such compensatory measures are only able to accommodate small increases in ICP. However, once compensatory mechanisms fail to control ICP then pressure rises unabated, leading to compression of blood vessels within the brain tissue, compromising tissue perfusion and leading to ischaemia, which further exacerbates cell injury and brain tissue dysfunction [76]. In an attempt maintain blood supply to the swollen brain, mean arterial blood pressure rises, leading to a further increase in ICP. Significantly elevated ICP opposes cerebral blood flow, adversely restricting blood perfusion to the brain, resulting in global ischemia and perpetuating tissue injury [65].

Persistently elevated ICP can force brain tissue to move down pressure gradients, in an attempt to relieve pressure, known as brain herniation. When the brain tissue herniates, it can compress adjacent structures causing dysfunction and blood vessels causing ischaemia, which further worsens tissue injury and lead to permanent brain tissue damage [77]. Life-threatening brain herniation occurs when the cerebellar tonsils herniate downwards through the foramen magnum, leading to compression of vital cardiorespiratory centres within the brain stem [78]. This can lead to intermittent or complete cessation of cardiorespiratory functions and subsequent death.

## 6. Current Treatments for Cerebral Oedema Following Acute CNS Injury

At present, there are a number of different approaches to the management of elevated ICP including head of bed elevation, transient hyperventilation, hypothermia, osmolar therapy (with mannitol or hypertonic saline (HTS)), barbituates and decompresssive surgery [78], of which the pharmacotherapies and surgical options will be discussed.

### 6.1. Osmolar Therapy

The most commonly used osmotic agents used to treat cerebral oedema and elevated ICP are mannitol and HTS [79]. Their predominate mechanism of action is believed to be the creation of an osmolar gradient which draws water from the brain, via rheological effects, into the intravascular space, thereby reducing total brain volume [70]. However, neuroprotection at the cellular level by osmotic agents has also been proposed, largely due to their ability to reduce oxidative stress and inflammation via cytokine-mediated pathways [80]. These agents are used in the management of cerebral oedema and elevated ICP despite a lack of high quality trails supporting their efficacy. Mannitol, an osmotic diuretic agent, is a commonly used front line agent in the management of elevated ICP, as it can be safely administered via a peripheral IV line [78]. Mannitol is frequently used in severely head injured patients, especially upon early presentation of increased ICP. Indeed, the majority of studies on the efficacy of mannitol therapy have been conducted in this cohort and generalised to other patient cohorts and there is little evidence for its use in the management of stroke patients with elevated ICP. The use of mannitol is contraindicated where there is clear evidence of BBB breakdown [81] and repeated dosing may lead to mannitol accumulation within the brain parenchyma, reversing the osmotic drive and increasing intra-parenchymal fluid accumulation, further elevating ICP and leading to neurological deterioration [70,82,83]. HTS is also used to manage elevated ICP. The dosing frequency and strategy of administration vary but reductions in ICP appear to be independent of the dosing regime. Despite this, treatment duration has not been adequately assessed and HTS treatment may be of limited efficacy in stroke patients when extensive BBB breakdown is prevalent, and may cause decreased platelet aggregation, prolonged coagulation and subsequent bleeding in addition to neurological ion imbalances including hypochloremia and hyperkalemia [84,85]. Each of the osmolar agents has its own inherent risks and complications, although HTS is associated with a reduced risk of ICP treatment failure [79] and may be used preferentially to mannitol in some patients, for example those with subarachnoid haemorrhage with vasopasm, where reduced volume depletion and hypotension may further compromise cerebral perfusion [78]. Furthermore, HTS is not a diuretic agent, which may be an advantage to mannitol in some patients. Regardless, both osmotherapies require monitoring of electrolytes, osmolarity and volume throughout treatment. Thus, osmotic agent therapy may be used a temporary measure to prevent acute brain stem compression until other measures such as decompressive surgery or haemorrhage evacuation can be performed [86]. However, osmotic agents do not target the mechanisms underlying abnormal fluid movement and accumulation within the brain parenchyma, they simply aim to reverse the osmotic drive and pull the excess fluid out of the brain tissue.

### 6.2. Barbituates

Barbiturates have been used to reduce ICP due to their ability to increase vascular tone, reduce cerebral metabolism and inhibit free radical-mediated lipid peroxidation [87,88]. Despitethis, barbiturate therapy does not interfere with the BBB disruption that underlies the genesis of cerebral oedema, which worsens over time following injury. However, barbituates are typically only used after other interventions have first been used, such as osmotic agents and sedation/mechanical ventilation and may be used in head injured patients to “put the brain to sleep”, reducing cerebral metabolism in an attempt to reduce further cerebral injury [78]. Indeed, barbiturate therapy is generally only used in extreme clinical situations as its use is associated with unsustained therapeutic properties and potential development of severe side effects, such as arterial hypotension and pulmonary failure [89,90]. Thus, the hypotensive effect of barbiturate therapy is likely to offset any reduction in ICP following treatment [91], although the anti-seizure activity of barbiturate therapy is a positive side effect of treatment [78].

### 6.3. Decompressive Craniectomy

Decompressive craniectomy (DC) aims to alleviate raised ICP through removal of part of the skull and opening of the underlying dura overlying the swollen brain to allow the brain to swell freely and reduce ICP [92]. The exact nature of the decompressive surgery and the location and amount of bone removed depends upon the underlying cause of the elevated ICP. In the setting of stroke, for example, malignant middle cerebral artery (MCA) infarction, typically a decompressive hemecraniectomy is performed where the skull overlying a hemisphere of the brain is removed. Whereas in TBI, for example in the case of diffuse axonal injury (DAI), large contusion or large intracranial haemorrhage, a bifrontal craniotomy is typically performed [78]. This surgical procedure is a physical measure only and does not influence the evolution of BBB structural/functional alterations or the genesis of cerebral oedema, which continue to worsen over time following injury. Nevertheless, DC has been demonstrated to be a life-saving procedure following both TBI and stroke [93,94], although this may be at the expense of increasing the number of patients who are severely disabled and dependent for tasks of daily living [93]. Overall, the functional outcome of the patient following decompressive surgery is ultimately determined by the severity of the cerebral insult, for example the brain injury or stroke, and the rehabilitation of the patient, important considerations for the patients neurosurgical team and family when making the decision to proceed with DC or not [78]. Although DC reduces ICP by providing additional space for the oedematous brain to swell, timing of the procedure is a crucial factor in determining outcome with DC surgery performed later than 24 h post-ictus is associated with a heightened mortality rate. Indeed, DC is typically performed after less invasive interventions such as osmotherapy, sedation and barbituates have been tried and ICP remains elevated [78]. Thus, early identification of evolving cerebral oedema is crucial in maximising treatment efficacy [95], as when DC is used only as a last resort after conservative physiological management and pharmacotherapy have failed to produce meaningful reduction in ICP, outcomes are predictably poor [96]. Furthermore, DC surgery is highly invasive with many contraindications for its use, in addition to the risks and potential complications including a higher mortality rate in those aged greater than 60 years of age and an increased likelihood of moderate to severe disability following surgery [97,98]. This becomes an issue of increasing concern, as stroke in particular is a disease associated with an ageing population, with an incidence in excess of 68% in those aged greater than 60 years of age [99]. The correlation between age and functional outcome remains an extremely important pre-treatment prognostic factor in deciding if patients should undergo DC [95].

### 6.4. Improving the Treatment of Cerebral Oedema and Elevated ICP

The burden of morbidity and mortality associated with cerebral oedema and elevated ICP, combined with and the inadequacy of current treatment interventions, emphasises that new treatment approaches are urgently required. It is clear that what all of the current treatments for cerebral oedema and elevated ICP have in common is that they do not target the underlying injury mechanisms which lead to increased BBB permeability and subsequent development of cerebral oedema and elevated ICP. There are currently no approved targeted treatments that interfere with these key injury processes to halt or reduce the evolution of BBB dysfunction and cerebral oedema following acute CNS injury. Indeed, to develop more effective treatments, it is essential to elucidate the processes underlying BBB disruption following acute CNS injury to allow the underlying mechanisms of cerebral oedema genesis to be targeted. Such an approach has the potential to markedly improve patient survival and outcome given that it is directly targeting the injury mechanisms and not just simply treating the symptoms of cerebral oedema and elevated ICP. Indeed, neurogenic inflammation, specifically the release of neuropeptides such as substance P (SP), has recently been shown to be involved in BBB permeability changes and development of cerebral oedema following acute CNS injury. Thus, directly targeting neurogenic inflammation in acute CNS injury may be a novel treatment strategy to reduce BBB permeability and in turn reduce the development of complications such as cerebral oedema and elevated ICP. Such an approach may provide enhanced treatment efficacy and duration compared with current treatment approaches.

## 7. Neurogenic Inflammation

The concept of neurogenic inflammation was first described in the peripheral nervous system (PNS), where activated neurons of the dorsal root ganglia were observed to induce blood vessel vasodilation in the lower extremities [100]. Following these early observations the definition of neurogenic inflammation has evolved to encompass a painful local inflammatory response characterized by vasodilation, increased vascular permeability, tissue swelling and mast cell degranulation [101]. In addition, there are tissue-specific responses such as smooth muscle contraction in the bladder, bronchoconstriction in the airways and ionotropic/chronotropic effects on the heart, amongst others [102]. Neurogenic inflammation may be initiated by a wide variety of agents including prostanoids, leukotrienes, histamine and serotonin, in addition to changes in the extracellular environment encompassing increased osmolarity, pH changes, heat, inflammation and mechanical stimulation [103,104].

The neuropeptide family is central to the development of neurogenic inflammation and comprises substance P (SP), calcitonin gene-related peptide (CGRP), neurokinin A (NKA), and neurokinin B (NKB), amongst others [101]. These neuropeptides act as neuromodulators and neurotransmitters, in both physiological and pathological processes [105]. In particular, SP is the most potent initiator of neurogenic inflammation, with CGRP able to further potentiate the effects of SP [103].

### 7.1. SP

SP is an 11 amino acid peptide member of the tackykinin peptide family that is produced from alternate splicing of the preprotachykinin a gene [106,107,108]. SP is released from primary afferent neurons, at both the central and peripheral nerve endings where it acts as a neurotransmitter. Under normal conditions, SP is synthesised and stored within both peripheral and central neurons [106,108,109], with activation or damage of these neurons resulting in the rapid release of SP [103,106]. SP may also be released from non-neuronal cells, including inflammatory and endothelial cells [106].

SP is widely distributed throughout both the CNS and PNS, localised in capsaicin-sensitive neurons and released in response to Ca^2+^-dependent depolarisation of neurons via various stimuli including, changes in pH, electrical stimulation and ligand binding, amongst others [103]. Within the CNS, the greatest SP immunoreactivity has been demonstrated in the amygdala, nucleus caudatus, putamen, globus pallidus, hypothalamus, substantia nigra and locus ceruleus [101,110]. Within the PNS, SP is found throughout the enteric nervous system, respiratory tract, urinary system, lymphoid organs, blood vessels and cellular components of the blood [101]. Indeed, SP-containing sensory nerves surround virtually all blood vessels in the body, with cerebral arteries having a particularly dense supply.

Following release, SP mediates its effects via high affinity binding to the neurokinin 1 (NK1) tachykinin receptor, but may also bind with varying affinity to the NK2 and NK3 tachykinin receptors depending upon receptor density/availability [111]. The NK receptors are members of the rhodopsin family of 7-transmembrane G-protein coupled receptors, with the G-proteins associated with the intracellular domain of the NK1 receptor responsible for the transduction of the SP signal. Stimulation of intracellular G proteins results in increased expression of cAMP and a cascade of events leading to regulation of ion channels, enzyme activity and changes in gene expression [111,112].

The NK1 tachykinin receptor is distributed throughout the CNS and is ubiquitously expressed throughout the brain [110]. Stimulation of the NK1 tachykinin receptor by SP initiates a number of biological processes including, vasodilation, smooth muscle contraction and relaxation, plasma protein extravasation, airway contraction [113]. In addition, roles for SP have been documented in nociception [114], learning and memory [115] and anxiety and depression [116]. SP has also been implicated in a number of different pathologies including migraine, anxiety, inflammatory bowel disease, and asthma, stroke [117,118,119] and TBI [46,120,121,122].

### 7.2. CGRP

CGRP is a 37 amino acid neuropeptide, co-expressed with neuropeptides such as SP in neuronal tissue [123]. It has two isoforms α-CGRP and β-CGRP, with α-CGRP more abundant in both the CNS [124] and PNS [125]. α-CGRP is formed from the alternative splicing of the calcitonin/CGRP gene located on chromosome 11. In particular, CGRP is highly expressed in all vascular tissues, the trigeminal ganglia and astroglial cells [126]. It is particularly active in the cerebral circulation [127] where it is stored and released from sensory neurons [128]. Calcitonin receptor-like receptor (CLR) is the receptor to which CGRP binds, comprised of two separate structures which come together, the G protein coupled receptor and CLR which is an accessory protein identified as receptor activity modifying protein (RAMP) [129]. The RAMP1 proteins are responsible for translocating the CLRs to the plasma membranes so that the CGRP molecules can bind to them. The CLR-RAMP1 receptor is expressed within the CNS endothelial cells. Upon binding to its receptor, CGRP activates the CLR-RAMP1 leading to increased cAMP levels which in turn cause potent vasodilation and increased blood flow [130,131], with such CGRP-mediated vessel dilation is located within the smooth muscle layer. CGRP has a particularly strong effect on cerebrovascular expansion [132], and given that it is co-stored and co-released with SP [103], it is involved in neurogenic inflammation and potentiating the effects of SP [133]. Beyond regulation of vascular tone, CGRP is also involved in angiogenesis, pain signalling and the regulation of different behavioural processes including the stress response and fear-related behaviours [133,134].

## 8. Neurogenic Inflammation in the CNS

Well documented in peripheral tissues [101], the concept of neurogenic inflammation as a response to tissue injury has more recently been extended to include the CNS [117,121,135]. Intravenous SP administration induces a significant increase in plasma extravasation in the dura mater, an effect abolished NK1 tachykinin receptor antagonist pre-treatment [136]. Furthermore, activation of NK1 tachykinin receptors on the vascular endothelium contributed to the development of cerebral oedema [135]. Similarly, treatment with capsaicin elicited a neurogenic inflammatory response within the dura mater [137]. Capsaicin-induced neuropeptide depletion was shown to provide protection from neonatal hypoxia/ischaemia injury in rats, resulting in reduction in infarct volume and apoptosis, and improved vascular dynamics, suggesting that neuropeptides were mediating such effects [138]. Indeed, initial studies in capsaicin pre-treated animals, to deplete sensory neuropeptides, have revealed that neuropeptide depletion prior to acute CNS injury is protective, with reductions in BBB permeability, cerebral oedema and both motor and cognitive deficits [121,139]. These studies clearly indicated a role for neuropeptides and neurogenic inflammation in these injury pathways. Subsequent studies have now clearly delineated a role for neurogenic inflammation in the BBB dysfunction and genesis of cerebral oedema observed following acute CNS injury [46,117,118,119,121,122]. In stroke, it is likely that the early development of cytotoxic oedema and associated intracellular swelling causes increases osmolality and nociceptor activation, in addition to the physical exertion of pressure on nerve fibres adjacent to engorged neurons are the stimuli which leads to their stimulation by mechanical means. Whereas in TBI, the mechanical shearing and laceration forces generated from movement of the brain within the skull at the moment of injury leads to neuropeptide release.

### 8.1. SP in TBI

Increased SP immunoreactivity has been observed in both human post-mortem tissue [140] and rodent TBI tissue [141]. Specifically, SP expression was increased at 5 h following rodent TBI in perivascular tissue and was present along blood vessels within the parenchyma [46,141], with such increases still evident at 24 h and 3 days post-TBI [142]. The increased perivascular SP was co-localised to regions of significant EB extravasation following TBI in rats, indicative of BBB disruption and vasogenic oedema [46]. Furthermore, increased SP was associated with persistent functional deficits [46,120].

### 8.2. SP in Stroke

Following observations of SP is release from the rabbit carotid body in response to hypoxia [143], suggesting that SP release is tissues response to hypoxia/ischemia, these findings have now been replicated in cerebral ischaemia [117,118,119,139,144]. Overexpression of SP has been observed following stroke, associated with an exacerbation of ischaemic tissue damage and poor neurological function [144]. Our group has since further explored the role of neurogenic inflammation in cerebral ischaemia [117,118,119,139]. We have shown that at 24 h following stroke with reperfusion, SP immunoreactivity was increased in penumbral tissue, but not within core tissue, of the infarcted hemisphere [117]. Such increases in SP immunoreactivity were observed in conjunction with significant disruption of the BBB, as measured by Evan’s Blue extravasation, in addition to profound cerebral oedema and persistent functional deficits [117,118,119,139]. A similar SP has been observed clinically with elevated SP levels shown to be present in the serum of patients with complete stroke or transient ischaemic attack (TIA) at 12–24 h following stroke onset [145]. Serum SP levels were on average four-fold higher in stroke/TIA patients comported with healthy controls at 12 h, which began to decline at 24 h following injury.

Taken together, these findings indicate that neurogenic inflammation, and in particular SP release, is a feature of acute CNS injury that appears be central to changes in BBB permeability and subsequent development of cerebral oedema, thereby providing a potential novel therapeutic target.

### 8.3. CGRP in TBI

CGRP has been implicated to be involved in the response to brain tissue damage [146] and various alterations in CGRP levels have been reported following trauma [147,148,149,150,151,152,153,154]. Following focal TBI in newborn piglets, a decrease in CGRP production was observed [147,148], a finding corroborated in focal murine TBI reporting decreased CGRP levels from 3 to 14 days post-trauma [153]. However, in direct contrast, focal rodent trauma lead to elevated plasma CGRP levels following trauma that peaked at 3 days and remained elevated at 7 days post-TBI [150]. Furthermore, CGRP levels are significantly elevated in the brain stem at 7, 14 and 28 days following focal murine trauma [149]. However, there are also reports increased serum levels of CGRP at 24 h, 3 h and 7 days following diffuse TBI [152], which was found to enhance fracture healing [151]. Such differences in the CGRP response to trauma may reflect the different patterns of injury between the various experimental trauma models, in addition to species-specific variations. Nevertheless, it appears that CGRP plays a role in clinical TBI as increased serum levels of CGRP were observed at 2 days following TBI in a clinical cohort [154].

### 8.4. CGRP in Stroke

Given its role as a potent vasodilator, it is not surprising that CGRP has been investigated in the setting of cerebral ischaemia as a potential neuroprotectant. Indeed, CGRP release increases under stressful conditions or ischaemic tissue damage [146].

This is in contrast to reports of reduced local CGRP levels following murine stroke with reperfusion, which is not conducive to the repair of damaged tissue [155]. However, leptin treatment was shown to enhance CGRP expression and in turn reduce infarct volume and both neuronal apoptosis and necrosis, whilst also improving regional CBF.

Furthermore, CGRP has been proposed as a modulator of post-stroke depression (PSD) following observations that both cerebrospinal fluid (CSF) and hippocampal levels of CGRP were elevated in a rodent PSD model [146]. Indeed, intracerebroventricular administration of CGRP enhanced PSD symptoms in a dose-dependent manner, suggesting that PSD is mediated, at least in part, by CGRP. A similar pattern has been observed in clinical depression with elevated CGRP levels recorded in the CSF of depressed patients [156].

Such findings indicate that alterations in CGRP levels following TBI and stroke are more varied than that of SP and are highly dependent upon the nature and severity of injury. Nevertheless, the increase in CGRP levels observed in some studies may represent a protective response to improve CBF and maintain tissue perfusion.

### 8.5. Neurogenic Inflammation and the BBB

It is clear that neurogenic inflammation increases BBB permeability and leads to downstream complications such as cerebral oedema. However, the exact mechanisms by which neurogenic inflammation, and specifically release of SP, leads to alterations in BBB permeability following acute CNS injury are unclear. Although some studies that have reported SP/NK1-induced alterations in BBB integrity and function. Application of SP to cerebral capillary endothelial cell cultures leads to a reduction in the expression of TJ components ZO-1 and caludin-5 but immediately following this TJs are observed to be intact [157]. In fact, it appears that SP may have its initial effects on transcellular transport across the BBB, specifically by increasing transcytosis via the activation of caveolae-mediated transport. Indeed, the NK1 tachykinin receptor is localised to caveolae within endothelial cells and upon stimulation can alter its expression or location, suggesting that it plays a dynamic role in this environment [158,159]. In keeping with this, SP-induced stimulation of the NK1 tachykinin receptor stimulates the relocation of protein kinase C-α to caveolae, a process integral to the internalisation of caveolae and therefore transcellular transport across the barrier [160].

However, the effects of SP on the BBB are not solely limited to alterations in permeability, with activation of the NK1 tachykinin receptor by SP increasing the migration of leukocytes, such as monocytes and neutrophils, via chemotactic effects [161,162,163], increased endothelial cell expression of adhesion molecules [164,165,166,167] and the exacerbation of local chemokine production [168]. Furthermore, SP applied to cultures of cerebral endothelial cells led to a dose-dependent increase in intracellular adhesion molecule-1, observed in conjunction with an increase in T cell adherence. Such findings suggest that increased SP levels in the setting of neurogenic inflammation has the capacity to increase the infiltration of inflammatory cells into the CNS tissue, in turn exacerbating the local neuroinflammatory response to acute CNS injury through the production of free radicals, pro-inflammatory cytokines and proteases such as MMPs [169]. Taken together, evidence suggests that neurogenic inflammation plays a role in BBB permeability changes but also has the capacity to perpetuate classical inflammation by enhancing immune cell trafficking into the brain, both of which lead to injury worsening. Given the role of the SP/NK1 system in alterations to BBB permeability and transport following acute CNS injury, modulating neurogenic inflammation may represent a novel treatment target to interfere with this key injury cascade.

## 9. Targeting Neurogenic Inflammation Following Acute CNS Injury

Many groups have hypothesised that modulating neurogenic inflammation may have therapeutic applications [116]. However, given the clear evidence that neurogenic inflammation is a feature of acute CNS injury associated with increased BBB permeability, genesis of cerebral oedema and the development of persistent functional deficits, it may represent a novel target for the treatment of cerebral oedema. NK1 tachykinin receptor antagonists are currently widely used clinically in patients undergoing chemotherapy to combat treatment nausea and are well tolerated [170,171]. However, their potential utility in the treatment of cerebral oedema following acute CNS injury has not been explored clinically.

### 9.1. NK1 Tachykinin Receptor Antagonists in TBI

Following observations that increased SP immunoreactivity was a feature of injury following brain trauma, NK1 tachykinin receptor antagonists have since been explored as potential therapeutic agents. Administration of the NK1 tachykinin receptor antagonist, *N*-acetyl-l-tryptophan (NAT), at 30 min post-TBI significantly reduced BBB permeability and levels of cerebral oedema, in addition to ameliorating functional deficits [46]. These findings have since been replicated, both in a focal TBI model [15] and in female animals [172]. Indeed, the therapeutic window for NK1 tachykinin receptor antagonist treatment is up to 12 h post-TBI, when a membrane-permeable form of the drug is used [120]. Beyond inhibition of neurogenic inflammation, the neuroprotective actions of SP blockade following trauma appear to be mediated, at least in part, by microglial inhibition [173]. Although these findings in rodent TBI models are extremely encouraging, given the extremely poor clinical translation from bench to bedside it is essential that such observations be verified in a large animal species to confirm efficacy before progressing to clinical assessment. Thus, we have since evaluated NK1 tachykinin receptor antagonist treatment in an ovine model of TBI [174], demonstrating a significant reduction ICP, with pressure returning to normal levels within 4 h of TBI.

### 9.2. NK1 Tachykinin Receptor Antagonists in Stroke

Given the similar nature of many secondary injury components following TBI and stroke, in particular BBB permeability alterations and subsequent development of cerebral oedema, it is not surprising that NK1 tachykinin receptor antagonist treatment has also proven beneficial following stroke. Yu and colleagues were the first to demonstrate that the NK1 tachykinin receptor antagonist SR-14033 reduced infarct volume and improved neurological function when measured at 24 h following focal cerebral ischaemia [144]. Despite these initial positive results, no further studies were conducted. Our group has since extensively characterised the effect of NK1 tachykinin receptor antagonist treatment following stroke. We have shown that NK1 tachykinin receptor antagonist treatment at 4 h post-stroke onset is associated with a significant reduction in BBB permeability and cerebral oedema, as measured at 24 h [118]. Furthermore, this was associated with a recovery of behavioural function to baseline levels within 4 days of stroke onset, although no effect on infarct volume was observed. Nevertheless, functional outcome is a more clinically useful measure of outcome. We have since demonstrated that NK1 tachykinin receptor antagonist treatment is effective in mild to severe strokes, with a therapeutic window of up to 8 h post-stroke for motor improvements and 12 h post-stroke for sensory function improvements [15]. Furthermore, we have demonstrated that NK1 tachykinin receptor antagonist treatment can safely and effectively be combined with thrombolysis with tPA [119]. NK1 tachykinin receptor antagonist treatment was not only more effective than either treatment alone, limiting the effects of reperfusion injury by reducing tPA-induced BBB permeability changes and intracerebral haemorrhage.

### 9.3. CGRP Agonists in Stroke

Given the potent vasodilatory actions of CGRP it is not surprising that it has been shown to have neuroprotective actions in conditions of ischaemia [175,176,177,178,179]. Increased CGRP immunoreactivity showed a positive correlation with tissue flap survival following ischaemia [175]. Despite this, investigations on CGRP treatment following stroke remain limited. Early CGRP intervention following brain injury can significantly reduce neuronal apoptosis and tissue damage whilst maintaining nerve regeneration [180].

CGRP induces dose-dependent increases in vasodilation post-ischaemia, enhancing reperfusion and potentially contributing to penumbral rescue [179]. Indeed, CGRP pre-treatment reduced infarct volume by 57% and significantly improved CBF in rodent stroke with reperfusion [181]. Furthermore, CGRP administered at the onset of reperfusion produced a significant reduction in infarct volume, BBB permeability and cerebral oedema following rodent stroke [178]. Such positive treatment effects were observed in concert with a reduction in expression of the water channel aquaporin 4, ultrastructural damage to endothelial cells and tight junction loss, suggesting that CGRP mediated stabilisation of the BBB. Indeed, exogenous CGRP significantly increased CBF and protected neurons following cerebral ischaemia [131]. CGRP has also proven beneficial in haemorrhagic stroke given its ability to counteract vasoconstriction in human subarachnoid haemorrhage (SAH) [182].

CGRP is central to the mechanisms underlying remote preconditioning, with intracerbroventricular morphine-induced remote preconditioning leading to increased CGRP release in a dose-dependent manner, suggesting that CGRP release was part of the protective response that reduced lesion volume to protect ischaemic brain tissue from ischaemia/reperfusion injury [183]. Such preconditioning effects are abolished when a CGRP release inhibitor is administered [184]. Delayed adrenomedullin (a member of the CGRP family) gene transfer 3 days following rodent stroke was associated with neuroprotection from the ischaemic insult, as evidenced by a reduction in infarct volume and apoptotic cell death combined with enhanced astrocyte migration [185].

There is a paucity of studies investigating the utility of CGRP agonists in TBI. However, given their beneficial role following stroke in salvaging ischaemic tissue, it seems feasible that they may also be effective following TBI, especially in the setting of injury progresses, cerebral oedema development and compromised cerebral perfusion.

## 10. Conclusions

It is clear that neurogenic inflammation is involved in enhanced permeability of the BBB following acute CNS injury and the subsequent development of cerebral oedema and poor outcomes. In particular, SP is a key player in these processes with NK1 tachykinin receptor antagonist treatment extremely effective in reducing BBB permeability, cerebral oedema and functional deficits in pre-clinical models of both TBI and stroke. The role of CGRP following acute CNS injury is less clear, with marked differences observed between injury models and severity. However, what is clear is that CGRP-induced vasodilation improves outcome in the setting of stroke and there is evidence that it stabilizes the BBB. CGRP agonists should be further explored for their potential utility in improving CBF and stabilising the BBB following acute CNS injury.

Given the inability of current treatments to target the mechanisms of BBB alterations and genesis of cerebral oedema that lead to elevations in ICP following acute CNS injury, modulation of neurogenic inflammation through the administration of an NK1 tachykinin receptor antagonist represents a novel therapeutic target to treat cerebral oedema to both reduce mortality and improve outcome. Furthermore, combination with a CGRP agonist may be an effective in modulating injury pathways in acute CNS injury. Thus, targeting neurogenic inflammation may provide an alternate treatment strategy that is more specific and efficacious than current pharmacotherapies used in the management of cerebral oedema and elevated ICP, all without the risk of invasive surgery.

## References

[1] Berkner J., Mannix R., Qiu J. (2016). Clinical traumatic brain injury in the preclinical setting. Methods Mol. Biol..

[2] Langlois J.A., Rutland-Brown W., Wald M.M. (2006). The epidemiology and impact of traumatic brain injury: A brief overview. J. Head Trauma Rehabil..

[3] Mackay J., Mensah G.A., Mendis S., Greenlund K. (2004). The Atlas of Heart Disease and Stroke.

[4] Feigin V.L., Forouzanfar M.H., Krishnamurthi R., Mensah G.A., Connor M., Bennett D.A., Moran A.E., Sacco R.L., Anderson L., Truelsen T. (2014). Global and regional burden of stroke during 1990–2010: Findings from the global burden of disease study 2010. Lancet.

[5] Janca A., Aarli J.A., Prilipko L., Dua T., Saxena S., Saraceno B. (2006). WHO/WFN Survey of neurological services: A worldwide perspective. J. Neurol. Sci..

[6] Asemota A.O., George B.P., Bowman S.M., Haider A.H., Schneider E.B. (2013). Causes and trends in traumatic brain injury for united states adolescents. J. Neurotrauma.

[7] Majdan M., Mauritz W., Brazinova A., Rusnak M., Leitgeb J., Janciak I., Wilbacher I. (2011). Severity and outcome of traumatic brain injuries (TBI) with different causes of injury. Brain Inj..

[8] Loane D.J., Faden A.I. (2010). Neuroprotection for traumatic brain injury: Translational challenges and emerging therapeutic strategies. Trends Pharmacol. Sci..

[9] Sims N.R., Anderson M.F. (2002). Mitochondrial contributions to tissue damage in stroke. Neurochem. Int..

[10] Hademenos G.J., Massoud T.F. (1997). Biophysical mechanisms of stroke. Stroke.

[11] Del Zoppo G.J., Hallenbeck J.M. (2000). Advances in the vascular pathophysiology of ischemic stroke. Thrombosis Res..

[12] Kwiatkowski T.G., Libman R.B., Frankel M., Tilley B.C., Morgenstern L.B., Lu M., Broderick J.P., Lewandowski C.A., Marler J.R., Levine S.R. (1999). Effects of tissue plasminogen activator for acute ischemic stroke at one year. National institute of neurological disorders and stroke recombinant tissue plasminogen activator stroke study group. N. Engl. J. Med..

[13] Ayata C., Ropper A.H. (2002). Ischaemic brain oedema. J. Clin. Neurosci..

[14] Muir K.W. (2013). Stroke. Medicine.

[15] Corrigan F., Vink R., Turner R.J. (2016). Inflammation in acute cns injury: A focus on the role of substance P. Br. J. Pharmacol..

[16] Ballabh P., Braun A., Nedergaard M. (2004). The blood-brain barrier: An overview: Structure, regulation, and clinical implications. Neurobiol. Dis..

[17] Begley D.J., Brightman M.W. (2003). Structural and functional aspects of the blood-brain barrier. Prog. Drug Res..

[18] Komarova Y., Malik A.B. (2010). Regulation of endothelial permeability via paracellular and transcellular transport pathways. Annu. Rev. Physiol..

[19] Parton R.G., Simons K. (2007). The multiple faces of caveolae. Nat. Rev. Mol. Cell Biol..

[20] Le Lay S., Kurzchalia T.V. (2005). Getting rid of caveolins: Phenotypes of caveolin-deficient animals. Biochim. Biophys. Acta.

[21] Schubert W., Frank P.G., Razani B., Park D.S., Chow C.W., Lisanti M.P. (2001). Caveolae-deficient endothelial cells show defects in the uptake and transport of albumin in vivo. J. Biol. Chem..

[22] Persidsky Y., Ramirez S.H., Haorah J., Kanmogne G.D. (2006). Blood-brain barrier: Structural components and function under physiologic and pathologic conditions. J. Neuroimmune Pharmacol..

[23] Joo F. (1993). The blood-brain barrier in vitro: The second decade. Neurochem. Int..

[24] Yang S., Jin H., Zhu Y., Wan Y., Zhu L., Hu B. (2017). Diverse functions and mechanisms of pericytes in ischemic stroke. Curr. Neuropharmacol..

[25] Lo E.H., Singhal A.B., Torchilin V.P., Abbott N.J. (2001). Drug delivery to damaged brain. Brain Res. Rev..

[26] Furuse M., Hirase T., Itoh M., Nagafuchi A., Yonemura S., Tsukita S., Tsukita S. (1993). Occludin: A novel integral membrane protein localizing at tight junctions. J. Cell Biol..

[27] Furuse M., Hata M., Furuse K., Yoshida Y., Haratake A., Sugitani Y., Noda T., Kubo A., Tsukita S. (2002). Claudin-based tight junctions are crucial for the mammalian epidermal barrier: A lesson from claudin-1-deficient mice. J. Cell Biol..

[28] Itoh M., Nagafuchi A., Yonemura S., Kitani-Yasuda T., Tsukita S., Tsukita S. (1993). The 220-kD protein colocalizing with cadherins in non-epithelial cells is identical to ZO-1, a tight junction-associated protein in epithelial cells: cDNA Cloning and immunoelectron microscopy. J. Cell Biol..

[29] Martin-Padura I., Lostaglio S., Schneemann M., Williams L., Romano M., Fruscella P., Panzeri C., Stoppacciaro A., Ruco L., Villa A. (1998). Junctional adhesion molecule, a novel member of the immunoglobulin superfamily that distributes at intercellular junctions and modulates monocyte transmigration. J. Cell Biol..

[30] Chen Y., Merzdorf C., Paul D.L., Goodenough D.A. (1997). COOH Terminus of occludin is required for tight junction barrier function in early xenopus embryos. J. Cell Biol..

[31] Medina R., Rahner C., Mitic L.L., Anderson J.M., van Itallie C.M. (2000). Occludin localization at the tight junction requires the second extracellular loop. J. Membr. Biol..

[32] Wong V., Gumbiner B.M. (1997). A synthetic peptide corresponding to the extracellular domain of occludin perturbs the tight junction permeability barrier. J. Cell Biol..

[33] Furuse M., Fujimoto K., Sato N., Hirase T., Tsukita S., Tsukita S. (1996). Overexpression of occludin, a tight junction-associated integral membrane protein, induces the formation of intracellular multilamellar bodies bearing tight junction-like structures. J. Cell Sci..

[34] Saitou M., Furuse M., Sasaki H., Schulzke J.D., Fromm M., Takano H., Noda T., Tsukita S. (2000). Complex phenotype of mice lacking occludin, a component of tight junction strands. Mol. Biol. Cell.

[35] Nitta T., Hata M., Gotoh S., Seo Y., Sasaki H., Hashimoto N., Furuse M., Tsukita S. (2003). Size-selective loosening of the blood-brain barrier in claudin-5-deficient mice. J. Cell Biol..

[36] Furuse M., Sasaki H., Fujimoto K., Tsukita S. (1998). A single gene product, claudin-1 or -2, reconstitutes tight junction strands and recruits occludin in fibroblasts. J. Cell Biol..

[37] Gonzalez-Mariscal L., Namorado M.C., Martin D., Luna J., Alarcon L., Islas S., Valencia L., Muriel P., Ponce L., Reyes J.L. (2000). Tight junction proteins ZO-1, ZO-2, and occludin along isolated renal tubules. Kidney Int..

[38] Fanning A.S., Jameson B.J., Jesaitis L.A., Anderson J.M. (1998). The tight junction protein ZO-1 establishes a link between the transmembrane protein occludin and the actin cytoskeleton. J. Biol. Chem..

[39] Hawkins B.T., Davis T.P. (2005). The blood-brain barrier/neurovascular unit in health and disease. Pharmacol. Rev..

[40] Jiao H., Wang Z., Liu Y., Wang P., Xue Y. (2011). Specific role of tight junction proteins claudin-5, occludin, and ZO-1 of the blood-brain barrier in a focal cerebral ischemic insult. J. Mol. Neurosci..

[41] Tornavaca O., Chia M., Dufton N., Almagro L.O., Conway D.E., Randi A.M., Schwartz M.A., Matter K., Balda M.S. (2015). ZO-1 controls endothelial adherens junctions, cell-cell tension, angiogenesis, and barrier formation. J. Cell Biol..

[42] Sifat A.E., Vaidya B., Abbruscato T.J. (2017). Blood-brain barrier protection as a therapeutic strategy for acute ischemic stroke. AAPS J..

[43] Preston E., Sutherland G., Finsten A. (1993). Three openings of the blood-brain barrier produced by forebrain ischemia in the rat. Neurosci. Lett..

[44] Donkin J.J., Vink R. (2010). Mechanisms of cerebral edema in traumatic brain injury: Therapeutic developments. Curr. Opin. Neurol..

[45] Prakash R., Carmichael S.T. (2015). Blood-brain barrier breakdown and neovascularization processes after stroke and traumatic brain injury. Curr. Opin. Neurol..

[46] Donkin J.J., Nimmo A.J., Cernak I., Blumbergs P.C., Vink R. (2009). Substance P is associated with the development of brain edema and functional deficits after traumatic brain injury. J. Cereb. Blood Flow Metab..

[47] Shlosberg D., Benifla M., Kaufer D., Friedman A. (2010). Blood-brain barrier breakdown as a therapeutic target in traumatic brain injury. Nat. Rev. Neurol..

[48] Shen W., Li S., Chung S.H., Zhu L., Stayt J., Su T., Couraud P.O., Romero I.A., Weksler B., Gillies M.C. (2011). Tyrosine phosphorylation of ve-cadherin and claudin-5 is associated with TGF-β1-induced permeability of centrally derived vascular endothelium. Eur. J. Cell Biol..

[49] Garcia C.M., Darland D.C., Massingham L.J., D’Amore P.A. (2004). Endothelial cell-astrocyte interactions and TGF β are required for induction of blood-neural barrier properties. Dev. Brain Res..

[50] Truettner J.S., Alonso O.F., Dietrich W.D. (2005). Influence of therapeutic hypothermia on matrix metalloproteinase activity after traumatic brain injury in rats. J. Cereb. Blood Flow Metab..

[51] Vilalta A., Sahuquillo J., Rosell A., Poca M.A., Riveiro M., Montaner J. (2008). Moderate and severe traumatic brain injury induce early overexpression of systemic and brain gelatinases. Intensive Care Med..

[52] Turner R.J., Sharp F.R. (2016). Implications of MMP9 for blood brain barrier disruption and hemorrhagic transformation following ischemic stroke. Front. Cell. Neurosci..

[53] Rosenberg G.A., Estrada E.Y., Dencoff J.E. (1998). Matrix metalloproteinases and timps are associated with blood-brain barrier opening after reperfusion in rat brain. Stroke.

[54] Gu Y., Zheng G., Xu M., Li Y., Chen X., Zhu W., Tong Y., Chung S.K., Liu K.J., Shen J. (2012). Caveolin-1 regulates nitric oxide-mediated matrix metalloproteinases activity and blood-brain barrier permeability in focal cerebral ischemia and reperfusion injury. J. Neurochem..

[55] Nag S., Venugopalan R., Stewart D.J. (2007). Increased caveolin-1 expression precedes decreased expression of occludin and claudin-5 during blood-brain barrier breakdown. Acta Neuropathol..

[56] Lakhan S.E., Kirchgessner A., Tepper D., Leonard A. (2013). Matrix metalloproteinases and blood-brain barrier disruption in acute ischemic stroke. Front. Neurol..

[57] Mun-Bryce S., Rosenberg G.A. (1998). Gelatinase B modulates selective opening of the blood-brain barrier during inflammation. Am. J. Phys..

[58] Asahi M., Wang X., Mori T., Sumii T., Jung J.C., Moskowitz M.A., Fini M.E., Lo E.H. (2001). Effects of matrix metalloproteinase-9 gene knock-out on the proteolysis of blood-brain barrier and white matter components after cerebral ischemia. J. Neurosci..

[59] Clark A.W., Krekoski C.A., Bou S.S., Chapman K.R., Edwards D.R. (1997). Increased gelatinase a (MMP-2) and gelatinase B (MMP-9) activities in human brain after focal ischemia. Neurosci. Lett..

[60] Anthony D.C., Ferguson B., Matyzak M.K., Miller K.M., Esiri M.M., Perry V.H. (1997). Differential matrix metalloproteinase expression in cases of multiple sclerosis and stroke. Neuropathol. Appl. Neurobiol..

[61] Montaner J., Molina C.A., Monasterio J., Abilleira S., Arenillas J.F., Ribo M., Quintana M., Alvarez-Sabin J. (2003). Matrix metalloproteinase-9 pretreatment level predicts intracranial hemorrhagic complications after thrombolysis in human stroke. Circulation.

[62] Ning M., Furie K.L., Koroshetz W.J., Lee H., Barron M., Lederer M., Wang X., Zhu M., Sorensen A.G., Lo E.H. (2006). Association between TPA therapy and raised early matrix metalloproteinase-9 in acute stroke. Neurology.

[63] Rosell A., Cuadrado E., Ortega-Aznar A., Hernandez-Guillamon M., Lo E.H., Montaner J. (2008). MMP-9-positive neutrophil infiltration is associated to blood-brain barrier breakdown and basal lamina type iv collagen degradation during hemorrhagic transformation after human ischemic stroke. Stroke.

[64] Hacke W.S.S., Horn M., Spranger M., de Georgia M., von Kummer R. (1996). “Malignant” middle cerebral artery territory infarction: Clinical course and prognostic signs. Arch. Neurol..

[65] Ropper A.H. (2014). Management of raised intracranial pressure and hyperosmolar therapy. Pract. Neurol..

[66] Treadwell S.D., Thanvi B. (2010). Malignant middle cerebral artery (MCA) infarction: Pathophysiology, diagnosis and management. Postgrad. Med. J..

[67] Battey T.W., Karki M., Singhal A.B., Wu O., Sadaghiani S., Campbell B.C., Davis S.M., Donnan G.A., Sheth K.N., Kimberly W.T. (2014). Brain edema predicts outcome after nonlacunar ischemic stroke. Stroke.

[68] Marmarou A. (2007). A review of progress in understanding the pathophysiology and treatment of brain edema. Neurosurg. Focus.

[69] Barros L.F., Castro J., Bittner C.X. (2002). Ion movements in cell death: From protection to execution. Biol. Res..

[70] Brogan M.E., Manno E.M. (2015). Treatment of malignant brain edema and increased intracranial pressure after stroke. Curr. Treat. Opt. Neurol..

[71] Simard J.M., Kent T.A., Chen M., Tarasov K.V., Gerzanich V. (2007). Brain oedema in focal ischaemia: Molecular pathophysiology and theoretical implications. Lancet Neurol..

[72] Michinaga S., Koyama Y. (2015). Pathogenesis of brain edema and investigation into anti-edema drugs. Int. J. Mol. Sci..

[73] Zhang S., He W.B., Chen N.H. (2014). Causes of death among persons who survive an acute ischemic stroke. Curr. Neurol. Neurosci. Rep..

[74] Hewitt A., Ellory C. (2012). Brain oedema, intracranial pressure and cerebral blood flow. Surgery.

[75] Treggiari M.M., Schutz N., Yanez N.D., Romand J.A. (2007). Role of intracranial pressure values and patterns in predicting outcome in traumatic brain injury: A systematic review. Neurocrit. Care.

[76] Jha S. (2003). Cerebral edema and its management. Med. J. Armed Forces India.

[77] Kim H., Jin S.T., Kim Y.W., Kim S.R., Park I.S., Jo K.W. (2015). Predictors of malignant brain edema in middle cerebral artery infarction observed on CT angiography. J. Clin. Neurosci..

[78] Freeman W.D. (2015). Management of intracranial pressure. Continuum.

[79] Burgess S., Abu-Laban R.B., Slavik R.S., Vu E.N., Zed P.J. (2016). A systematic review of randomized controlled trials comparing hypertonic sodium solutions and mannitol for traumatic brain injury: Implications for emergency department management. Ann. Pharmacother..

[80] Mojtahedzadeh M., Ahmadi A., Mahmoodpoor A., Beigmohammadi M.T., Abdollahi M., Khazaeipour Z., Shaki F., Kuochaki B., Hendouei N. (2014). Hypertonic saline solution reduces the oxidative stress responses in traumatic brain injury patients. J. Res. Med. Sci..

[81] Witherspoon B., Ashby N.E. (2017). The use of mannitol and hypertonic saline therapies in patients with elevated intracranial pressure: A review of the evidence. Nurs. Clin. N. Am..

[82] Jaeman Cho Y.-H.K., Soo Han H., Park J. (2007). Accumulated mannitol and aggravated cerebral edema in a rat model of middle cerebral artery infarction. J. Korean Neurosurg. Soc..

[83] Maioriello A.V., Chaljub G., Nauta H.J., Lacroix M. (2002). Chemical shift imaging of mannitol in acute cerebral ischemia. Case report. J. Neurosurg..

[84] Hauer E.M., Stark D., Staykov D., Steigleder T., Schwab S., Bardutzky J. (2011). Early continuous hypertonic saline infusion in patients with severe cerebrovascular disease. Crit. Care Med..

[85] Surani S., Lockwood G., Macias M.Y., Guntupalli B., Varon J. (2015). Hypertonic saline in elevated intracranial pressure: Past, present, and future. J. Intensive Care Med..

[86] Grande P.O., Romner B. (2012). Osmotherapy in brain edema: A questionable therapy. J. Neurosurg. Anesthesiol..

[87] Demopoulos H.B., Flamm E.S., Pietronigro D.D., Seligman M.L. (1980). The free radical pathology and the microcirculation in the major central nervous system disorders. Acta Phys. Scand. Suppl..

[88] Kassell N.F., Peerless S.J., Drake C.G., Boarini D.J., Adams H.P. (1980). Treatment of ischemic deficits from cerebral vasospasm with high dose barbiturate therapy. Neurosurgery.

[89] Bardutzky J., Schwab S. (2007). Antiedema therapy in ischemic stroke. Stroke.

[90] Schwab S., Spranger M., Schwarz S., Hacke W. (1997). Barbiturate coma in severe hemispheric stroke: Useful or obsolete?. Neurology.

[91] Roberts I., Sydenham E. (2012). Barbiturates for acute traumatic brain injury. Cochrane Database Syst. Rev..

[92] Yang X.F., Yao Y., Hu W.W., Li G., Xu J.F., Zhao X.Q., Liu W.G. (2005). Is decompressive craniectomy for malignant middle cerebral artery infarction of any worth?. J. Zhejiang Univ. Sci. B.

[93] Jaeger M., Soehle M., Meixensberger J. (2003). Effects of decompressive craniectomy on brain tissue oxygen in patients with intracranial hypertension. J. Neurol. Neurosurg. Psychiatry.

[94] Mori K., Aoki A., Yamamoto T., Horinaka N., Maeda M. (2001). Aggressive decompressive surgery in patients with massive hemispheric embolic cerebral infarction associated with severe brain swelling. Acta Neurochir..

[95] Chen C.C., Cho D.Y., Tsai S.C. (2007). Outcome of and prognostic factors for decompressive hemicraniectomy in malignant middle cerebral artery infarction. J. Clin. Neurosci..

[96] Grindlinger G.A., Skavdahl D.H., Ecker R.D., Sanborn M.R. (2016). Decompressive craniectomy for severe traumatic brain injury: Clinical study, literature review and meta-analysis. Springerplus.

[97] Hofmeijer J., Kappelle L.J., Algra A., Amelink G.J., van Gijn J., van der Worp H.B. (2009). Surgical decompression for space-occupying cerebral infarction (the hemicraniectomy after middle cerebral artery infarction with life-threatening edema trial [HAMLET]): A multicentre, open, randomised trial. Lancet Neurol..

[98] Vahedi K., Hofmeijer J., Juettler E., Vicaut E., George B., Algra A., Amelink G.J., Schmiedeck P., Schwab S., Rothwell P.M. (2007). Early decompressive surgery in malignant infarction of the middle cerebral artery: A pooled analysis of three randomised controlled trials. Lancet Neurol..

[99] Mozaffarian D., Benjamin E.J., Go A.S., Arnett D.K., Blaha M.J., Cushman M., de Ferranti S., Despres J.P., Fullerton H.J., Howard V.J. (2015). Heart disease and stroke statistics—2015 update: A report from the american heart association. Circulation.

[100] Bayliss W. (1901). On the origin from the spinal cord of the vaso-dilator fibres of the hind-limb, and on the nature of these fibres. J. Physiol..

[101] Severini C., Improta G., Falconieri-Erspamer G., Salvadori S., Erspamer V. (2002). The tachykinin peptide family. Pharmacol. Rev..

[102] Black P.H. (2002). Stress and the inflammatory response: A review of neurogenic inflammation. Brain Behav. Immun..

[103] Harrison S., Geppetti P. (2001). Substance P. Int. J. Biochem. Cell Biol..

[104] Lewis K.M., Turner R.J., Vink R. (2013). Blocking neurogenic inflammation for the treatment of acute disorders of the central nervous system. Int. J. Inflam..

[105] Kleinman J.E., Hong J., Iadarola M., Govoni S., Gillin C.J. (1985). Neuropeptides in human brain—Postmortem studies. Prog. Neuro Psychopharmacol. Biol. Psychiatry.

[106] Hokfelt T., Broberger C., Xu Z.Q., Sergeyev V., Ubink R., Diez M. (2000). Neuropeptide—An overview. Neuropharmacology.

[107] Leeman S.E., Ferguson S.L. (2000). Substance P: An historical perspective. Neuropeptides.

[108] Maggi C.A. (1995). The mammalian tachykinin receptors. Gen. Pharmacol..

[109] Otsuka M., Yoshioka K. (1993). Neurotransmitter functions of mammalian tachykinins. Phys. Rev..

[110] Sutoo D., Yabe K., Akiyama K. (1999). Quantitative imaging of substance P in the human brain using a brain mapping analyzer. Neurosci. Res..

[111] Regoli D., Boudon A., Fauchere J.L. (1994). Receptors and antagonists for substance P and related peptides. Pharmacol. Rev..

[112] Kavelaars A., Broeke D., Jeurissen F., Kardux J., Meijer A., Franklin R., Gelfand E.W., Heijnen C.J. (1994). Activation of human monocytes via a non-neurokinin substance P receptor that is coupled to GI protein, calcium, phospholipase D, map kinase, and IL-6 production. J. Immunol..

[113] Campos M.M., Calixto J.B. (2000). Neurokinin mediation of edema and inflammation. Neuropeptides.

[114] Wu H.E., Schwasinger E.T., Hong J.S., Tseng L.F. (2005). Pretreatment with antiserum against dynorphin, substance P, or cholecystokinin enhances the morphine-produced anti-allodynia in the sciatic nerve ligated mice. Neurosci. Lett..

[115] Hasenohrl R.U., Souza-Silva M.A., Nikolaus S., Tomaz C., Brandao M.L., Schwarting R.K., Huston J.P. (2000). Substance P and its role in neural mechanisms governing learning, anxiety and functional recovery. Neuropeptides.

[116] Rupniak N.M., Kramer M.S. (1999). Discovery of the antidepressant and anti-emetic efficacy of substance P receptor (NK1) antagonists. Trends Pharmacol. Sci..

[117] Turner R.J., Blumbergs P.C., Sims N.R., Helps S.C., Rodgers K.M., Vink R. (2006). Increased substance p immunoreactivity and edema formation following reversible ischemic stroke. Acta Neurochir. Suppl..

[118] Turner R.J., Helps S.C., Thornton E., Vink R. (2011). A substance P antagonist improves outcome when administered 4 h after onset of ischaemic stroke. Brain Res..

[119] Turner R.J., Vink R. (2012). Combined tissue plasminogen activator and an NK1 tachykinin receptor antagonist: An effective treatment for reperfusion injury following acute ischemic stroke in rats. Neuroscience.

[120] Donkin J.J., Cernak I., Blumbergs P.C., Vink R. (2011). A substance P antagonist reduces axonal injury and improves neurologic outcome when administered up to 12 h after traumatic brain injury. J. Neurotrauma.

[121] Nimmo A.J., Cernak I., Heath D.L., Hu X., Bennett C.J., Vink R. (2004). Neurogenic inflammation is associated with development of edema and functional deficits following traumatic brain injury in rats. Neuropeptides.

[122] Vink R., Donkin J.J., Cruz M.I., Nimmo A.J., Cernak I. (2004). A substance P antagonist increases brain intracellular free magnesium concentration after diffuse traumatic brain injury in rats. J. Am. Coll. Nutr..

[123] Ohtori S., Takahashi K., Chiba T., Yamagata M., Sameda H., Moriya H. (2002). Substance P and calcitonin gene-related peptide immunoreactive sensory drg neurons innervating the lumbar intervertebral discs in rats. Ann. Anat. Anat. Anz..

[124] Lange M., Enkhbaatar P., Traber D.L., Cox R.A., Jacob S., Mathew B.P., Hamahata A., Traber L.D., Herndon D.N., Hawkins H.K. (2009). Role of calcitonin gene-related peptide (CGRP) in ovine burn and smoke inhalation injury. J. Appl. Physiol..

[125] Rosenfeld M.G., Mermod J.J., Amara S.G., Swanson L.W., Sawchenko P.E., Rivier J., Vale W.W., Evans R.M. (1983). Production of a novel neuropeptide encoded by the calcitonin gene via tissue-specific RNA processing. Nature.

[126] Moreno M.J., Cohen Z., Stanimirovic D.B., Hamel E. (1999). Functional calcitonin gene-related peptide type 1 and adrenomedullin receptors in human trigeminal ganglia, brain vessels, and cerebromicrovascular or astroglial cells in culture. J. Cereb. Blood Flow Metab..

[127] Hanko J., Hardebo J.E., Kahrstrom J., Owman C., Sundler F. (1985). Calcitonin gene-related peptide is present in mammalian cerebrovascular nerve fibres and dilates pial and peripheral arteries. Neurosci. Lett..

[128] Amara S.G., Jonas V., Rosenfeld M.G., Ong E.S., Evans R.M. (1982). Alternative RNA processing in calcitonin gene expression generates mRNAs encoding different polypeptide products. Nature.

[129] McLatchie L.M., Fraser N.J., Main M.J., Wise A., Brown J., Thompson N., Solari R., Lee M.G., Foord S.M. (1998). Ramps regulate the transport and ligand specificity of the calcitonin-receptor-like receptor. Nature.

[130] Bulloch K., Milner T.A., Prasad A., Hsu M., Buzsaki G., McEwen B.S. (1998). Induction of calcitonin gene-related peptide-like immunoreactivity in hippocampal neurons following ischemia: A putative regional modulator of the CNS injury/immune response. Exp. Neurol..

[131] Zhang Z.H., Fang X.B., Xi G.M., Li W.C., Ling H.Y., Qu P. (2010). Calcitonin gene-related peptide enhances creb phosphorylation and attenuates tau protein phosphorylation in rat brain during focal cerebral ischemia/reperfusion. Biomed. Pharmacother..

[132] Omeis I., Neil J.A., Murali R., Abrahams J.M. (2008). Treatment of cerebral vasospasm with biocompatible controlled-release systems for intracranial drug delivery. Neurosurgery.

[133] Legat F.J., Griesbacher T., Schicho R., Althuber P., Schuligoi R., Kerl H., Wolf P. (2002). Repeated subinflammatory ultraviolet b irradiation increases substance P and calcitonin gene-related peptide content and augments mustard oil-induced neurogenic inflammation in the skin of rats. Neurosci. Lett..

[134] Oku R., Satoh M., Fujii N., Otaka A., Yajima H., Takagi H. (1987). Calcitonin gene-related peptide promotes mechanical nociception by potentiating release of substance P from the spinal dorsal horn in rats. Brain Res..

[135] Stumm R., Culmsee C., Schafer M.K., Krieglstein J., Weihe E. (2001). Adaptive plasticity in tachykinin and tachykinin receptor expression after focal cerebral ischemia is differentially linked to gabaergic and glutamatergic cerebrocortical circuits and cerebrovenular endothelium. J. Neurosci..

[136] Cyrino L.A., Cardoso R.C., Hackl L.P., Nicolau M. (2002). Effect of quercetin on plasma extravasation in rat CNS and dura mater by ACE and NEP inhibition. Phytother. Res..

[137] Markowitz S., Saito K., Moskowitz M.A. (1987). Neurogenically mediated leakage of plasma protein occurs from blood vessels in dura mater but not brain. J. Neurosci..

[138] Khatibi N.H., Jadhav V., Charles S., Chiu J., Buchholz J., Tang J., Zhang J.H. (2011). Capsaicin pre-treatment provides neurovascular protection against neonatal hypoxic-ischemic brain injury in rats. Acta Neurochir. Suppl..

[139] Turner R.J., Vink R. (2014). NK1 Tachykinin receptor treatment is superior to capsaicin pre-treatment in improving functional outcome following acute ischemic stroke. Neuropeptides.

[140] Zacest A.C., Vink R., Manavis J., Sarvestani G.T., Blumbergs P.C. (2010). Substance P immunoreactivity increases following human traumatic brain injury. Acta Neurochir. Suppl..

[141] Donkin J.J., Turner R.J., Hassan I., Vink R. (2007). Substance P in traumatic brain injury. Prog. Brain Res..

[142] Cook N.L., Vink R., Donkin J.J., van den Heuvel C. (2009). Validation of reference genes for normalization of real-time quantitative RT-PCR data in traumatic brain injury. J. Neurosci. Res..

[143] Kim D.K., Oh E.K., Summers B.A., Prabhakar N.R., Kumar G.K. (2001). Release of substance P by low oxygen in the rabbit carotid body: Evidence for the involvement of calcium channels. Brain Res..

[144] Yu Z., Cheng G., Huang X., Li K., Cao X. (1997). Neurokinin-1 receptor antagonist sr140333: A novel type of drug to treat cerebral ischemia. Neuroreport.

[145] Bruno G., Tega F., Bruno A., Graf U., Corelli F., Molfetta R., Barucco M. (2003). The role of substance P in cerebral ischemia. Int. J. Immunopathol. Pharmacol..

[146] Shao B., Zhou Y.L., Wang H., Lin Y.S. (2015). The role of calcitonin gene-related peptide in post-stroke depression in chronic mild stress-treated ischemic rats. Phys. Behav..

[147] Armstead W.M. (2000). Age-dependent cerebral hemodynamic effects of traumatic brain injury in newborn and juvenile pigs. Microcirculation.

[148] Armstead W.M., Vavilala M.S. (2007). Adrenomedullin reduces gender-dependent loss of hypotensive cerebrovasodilation after newborn brain injury through activation of ATP-dependent k channels. J. Cereb. Blood Flow Metab..

[149] Elliott M.B., Oshinsky M.L., Amenta P.S., Awe O.O., Jallo J.I. (2012). Nociceptive neuropeptide increases and periorbital allodynia in a model of traumatic brain injury. Headache.

[150] Hang C.H., Shi J.X., Li J.S., Wu W., Li W.Q., Yin H.X. (2004). Levels of vasoactive intestinal peptide, cholecystokinin and calcitonin gene-related peptide in plasma and jejunum of rats following traumatic brain injury and underlying significance in gastrointestinal dysfunction. World J. Gastroenterol..

[151] Song Y., Bi L., Zhang Z., Huang Z., Hou W., Lu X., Sun P., Han Y. (2012). Increased levels of calcitonin gene-related peptide in serum accelerate fracture healing following traumatic brain injury. Mol. Med. Rep..

[152] Song Y., Han G.X., Chen L., Zhai Y.Z., Dong J., Chen W., Li T.S., Zhu H.Y. (2017). The role of the hippocampus and the function of calcitonin gene-related peptide in the mechanism of traumatic brain injury accelerating fracture-healing. Eur. Rev. Med. Pharmacol. Sci..

[153] Wang Z., Wang Q., Wang C., Xu X., Yu H. (2017). Tetramethylpyrazine attenuates periorbital allodynia and neuroinflammation in a model of traumatic brain injury. J. Inflamm..

[154] Yang X., Shi Z., Li X., Li J. (2015). Impacts of stellate ganglion block on plasma NF-κB and inflammatory factors of TBI patients. Int. J. Clin. Exp. Med..

[155] Zhang J.Y., Yan G.T., Liao J., Deng Z.H., Xue H., Wang L.H., Zhang K. (2011). Leptin attenuates cerebral ischemia/reperfusion injury partially by cgrp expression. Eur. J. Pharmacol..

[156] Mathe A.A., Agren H., Lindstrom L., Theodorsson E. (1994). Increased concentration of calcitonin gene-related peptide in cerebrospinal fluid of depressed patients. A possible trait marker of major depressive disorder. Neurosci. Lett..

[157] Lu T.S., Avraham H.K., Seng S., Tachado S.D., Koziel H., Makriyannis A., Avraham S. (2008). Cannabinoids inhibit HIV-1 GP120-mediated insults in brain microvascular endothelial cells. J. Immunol..

[158] Kubale V., Abramovic Z., Pogacnik A., Heding A., Sentjurc M., Vrecl M. (2007). Evidence for a role of caveolin-1 in neurokinin-1 receptor plasma-membrane localization, efficient signaling, and interaction with β-arrestin 2. Cell Tissue Res..

[159] Monastyrskaya K., Hostettler A., Buergi S., Draeger A. (2005). The NK1 receptor localizes to the plasma membrane microdomains, and its activation is dependent on lipid raft integrity. J. Biol. Chem..

[160] Mineo C., Ying Y.S., Chapline C., Jaken S., Anderson R.G. (1998). Targeting of protein kinase calpha to caveolae. J. Cell Biol..

[161] Cao T., Pinter E., Al-Rashed S., Gerard N., Hoult J.R., Brain S.D. (2000). Neurokinin-1 receptor agonists are involved in mediating neutrophil accumulation in the inflamed, but not normal, cutaneous microvasculature: An in vivo study using neurokinin-1 receptor knockout mice. J. Immunol..

[162] Schratzberger P., Reinisch N., Prodinger W.M., Kahler C.M., Sitte B.A., Bellmann R., Fischer-Colbrie R., Winkler H., Wiedermann C.J. (1997). Differential chemotactic activities of sensory neuropeptides for human peripheral blood mononuclear cells. J. Immunol..

[163] Souza D.G., Mendonca V.A., de A Castro M.A., Poole S., Teixeira M.M. (2002). Role of tachykinin NK receptors on the local and remote injuries following ischaemia and reperfusion of the superior mesenteric artery in the rat. Br. J. Pharmacol..

[164] Annunziata P., Cioni C., Santonini R., Paccagnini E. (2002). Substance P antagonist blocks leakage and reduces activation of cytokine-stimulated rat brain endothelium. J. Neuroimmunol..

[165] Li P.C., Chen W.C., Chang L.C., Lin S.C. (2008). Substance P acts via the neurokinin receptor 1 to elicit bronchoconstriction, oxidative stress, and upregulated icam-1 expression after oil smoke exposure. Am. J. Phys. Lung Cell. Mol. Phys..

[166] Toneatto S., Finco O., van der Putten H., Abrignani S., Annunziata P. (1999). Evidence of blood-brain barrier alteration and activation in HIV-1 GP120 transgenic mice. AIDS.

[167] Vishwanath R., Mukherjee R. (1996). Substance P promotes lymphocyte-endothelial cell adhesion preferentially via LFA-1/ICAM-1 interactions. J. Neuroimmunol..

[168] Ramnath R.D., Bhatia M. (2006). Substance P treatment stimulates chemokine synthesis in pancreatic acinar cells via the activation of NF-κB. Am. J. Phys. Gastrointest. Liver Phys..

[169] Corrigan F., Mander K.A., Leonard A.V., Vink R. (2016). Neurogenic inflammation after traumatic brain injury and its potentiation of classical inflammation. J. Neuroinflamm..

[170] Huskey S.E., Dean B.J., Bakhtiar R., Sanchez R.I., Tattersall F.D., Rycroft W., Hargreaves R., Watt A.P., Chicchi G.G., Keohane C. (2003). Brain penetration of aprepitant, a substance P receptor antagonist, in ferrets. Drug Metab. Dispos..

[171] Inoue T., Kimura M., Uchida J., Nishino K., Kumagai T., Taniguchi J., Imamura F. (2017). Aprepitant for the treatment of breakthrough chemotherapy-induced nausea and vomiting in patients receiving moderately emetogenic chemotherapy. Int. J. Clin. Oncol..

[172] Corrigan F., Leonard A., Ghabriel M., van den Heuvel C., Vink R. (2012). A substance P antagonist improves outcome in female sprague dawley rats following diffuse traumatic brain injury. CNS Neurosci. Ther..

[173] Carthew H.L., Ziebell J.M., Vink R. (2012). Substance P-induced changes in cell genesis following diffuse traumatic brain injury. Neuroscience.

[174] Gabrielian L., Helps S.C., Thornton E., Turner R.J., Leonard A.V., Vink R. (2013). Substance P antagonists as a novel intervention for brain edema and raised intracranial pressure. Acta Neurochir. Suppl..

[175] Bucinskaite V., Brodda-Jansen G., Stenfors C., Theodorsson E., Lundeberg T. (1998). Increased concentrations of calcitonin gene-related peptide-like immunoreactivity in rat brain and peripheral tissue after ischaemia: Correlation to flap survival. Neuropeptides.

[176] Cai H., Xu X., Liu Z., Wang Q., Feng G., Li Y., Xu C., Liu G., Li Z. (2010). The effects of calcitonin gene-related peptide on BFGF and AQP4 expression after focal cerebral ischemia reperfusion in rats. Pharmazie.

[177] Kjartansson J., Dalsgaard C.J. (1987). Calcitonin gene-related peptide increases survival of a musculocutaneous critical flap in the rat. Eur. J. Pharmacol..

[178] Liu Z., Liu Q., Cai H., Xu C., Liu G., Li Z. (2011). Calcitonin gene-related peptide prevents blood-brain barrier injury and brain edema induced by focal cerebral ischemia reperfusion. Regul. Pept..

[179] Tam C., Brain S.D. (2004). The assessment of vasoactive properties of cgrp and adrenomedullin in the microvasculature: A study using in vivo and in vitro assays in the mouse. J. Mol. Neurosci..

[180] Macdonald R.L., Pluta R.M., Zhang J.H. (2007). Cerebral vasospasm after subarachnoid hemorrhage: The emerging revolution. Nat. Clin. Pract. Neurol..

[181] Holland J.P., Sydserff S.G., Taylor W.A., Bell B.A. (1994). Calcitonin gene-related peptide reduces brain injury in a rat model of focal cerebral ischemia. Stroke.

[182] Juul R., Aakhus S., Bjornstad K., Gisvold S.E., Brubakk A.O., Edvinsson L. (1994). Calcitonin gene-related peptide (human α-CGRP) counteracts vasoconstriction in human subarachnoid haemorrhage. Neurosci. Lett..

[183] Zhang Y., Irwin M.G., Lu Y., Mei B., Zuo Y.M., Chen Z.W., Wong T.M. (2011). Intracerebroventricular administration of morphine confers remote cardioprotection—Role of opioid receptors and calmodulin. Eur. J. Pharmacol..

[184] Rehni A.K., Singh T.G., Jaggi A.S., Singh N. (2008). Pharmacological preconditioning of the brain: A possible interplay between opioid and calcitonin gene related peptide transduction systems. Pharmacol. Rep..

[185] Xia C.F., Yin H., Borlongan C.V., Chao J., Chao L. (2004). Adrenomedullin gene delivery protects against cerebral ischemic injury by promoting astrocyte migration and survival. Hum. Gene Ther..

